# Endocytic BDNF secretion regulated by Vamp3 in astrocytes

**DOI:** 10.1038/s41598-021-00693-w

**Published:** 2021-10-27

**Authors:** Jeongho Han, Sungryeong Yoon, Hyungju Park

**Affiliations:** 1 Research Group of Neurovascular Unit, Korea Brain Research Institute (KBRI), Daegu, 41062 South Korea; 2 Department of Brain and Cognitive Sciences, DGIST, Daegu, 42988 South Korea

**Keywords:** Astrocyte, Endosomes, Exocytosis, Cellular neuroscience, Glial biology, Neurotrophic factors

## Abstract

Brain-derived neurotrophic factor (BDNF) regulates diverse brain functions via TrkB receptor signaling. Due to the expression of TrkB receptors, astrocytes can internalize extracellular BDNF proteins via receptor-mediated endocytosis. Endocytosed BDNF can be re-secreted upon stimulation, but the molecular mechanism underlying this phenomenon remains unrecognized. Our study reveals that vesicle-associated membrane protein 3 (Vamp3) selectively regulates the release of endocytic BDNF from astrocytes. By using quantum dot (QD)-conjugated mature BDNF (QD-BDNF) as a proxy for the extracellular BDNF protein, we monitored the uptake, transport, and secretion of BDNF from cultured cortical astrocytes. Our data showed that endocytic QD-BDNF particles were enriched in Vamp3-containing vesicles in astrocytes and that ATP treatment sufficiently triggered either the antero- or retrograde transport and exocytosis of QD-BDNF-containing vesicles. Downregulation of Vamp3 expression disrupted endocytic BDNF secretion from astrocytes but did not affect uptake or transport. Collectively, these results provide evidence of the selective ability of astrocytic Vamp3 to control endocytic BDNF secretion during BDNF recycling.

## Introduction

Brain-derived neurotrophic factor (BDNF) regulates diverse brain functions, including cell survival, differentiation, synaptic connectivity, and cognitive processes^1–3^. Secretion of either the pro-form of BDNF (proBDNF) or the mature form of BDNF (mBDNF) from dense-core vesicles depends on the Ca^2+^-mediated actions of vesicular exocytosis machineries such as *S*oluble *N*SF *A*ttachment protein *Re*ceptor (SNARE) proteins^4,5^. Extracellular proBDNF and mBDNF bind to pan-neurotrophin receptor p75 (p75NTR) and tropomyosin-related kinase B (TrkB), respectively^2^ and can reside in endosomal compartments in nearby target cells after receptor-mediated endocytosis. While the BDNF-TrkB complex in neuronal endosomes can be retrogradely transported or remain active in the form of a “signaling endosome”, extracellular BDNF can also be recycled by re-secretion in response to neuronal activity^6–8^.

Astrocytes are also thought to recycle extracellular BDNF proteins. ProBDNF was shown to be internalized through p75NTR-dependent endocytosis, and this endocytosed neuronal proBDNF appeared to be re-secreted as mBDNF^9,10^. The maintenance of long-term potentiation (LTP) and memory acquisition requires the astrocytic secretion of endocytic BDNF^9,10^. On the other hand, mBDNF seems to be absorbed by astrocytes due to their strong expression of TrkB^11,12^; however, the re-secretion of endocytic mBDNF has not yet been directly assessed. Neurons require complexin-1/2 and synaptotagmin 6 for the activity-dependent re-secretion of endocytic mBDNF^7^, but the molecular mechanisms underlying the recycling of mBDNF in astrocytes are unknown.

Astrocytes respond to neurotransmitters or active substances, such as glutamate and ATP, displaying the increase in the intracellular Ca^2+^ concentration through the activation of corresponding receptors^13,14^ Because vesicular exocytosis is dependent on Ca^2+^-dependent SNARE proteins, the astrocytic Ca^2+^-dependent actions of SNARE machinery could feasibly be involved in the release of mBDNF from astrocytes. Among SNARE machinery proteins expressed in astrocytes, such as vesicle-associated membrane proteins 2, 3, and 7 (Vamp2, Vamp3, and Vamp7, respectively)^15^, the role of Vamp3 in regulating endocytic BDNF secretion is of interest because of its role in endosome recycling^16^. In this study, we provide evidence that Vamp3 is one of primary mechanisms controlling endocytic mBDNF release from astrocytes. Monitoring the direct uptake, transport and ATP-dependent exocytosis of endocytic mBDNF in astrocytes utilizing recombinant mBDNF proteins linked to quantum dots (QDs) revealed that Vamp3 was selectively involved in the exocytosis of endocytic mBDNF. Our QD-linked mBDNF sensor was sufficient for examining the transport and activity-dependent secretion of endosomes, as reported previously^7,17,18^, due to the excellent photostability and high signal-to-noise ratio of QDs in live cells. These results support the notion that mBDNF recycling in astrocytes serves as an additional source of extracellular BDNF, which is crucial for activity-dependent synaptic plasticity.

## Methods

Detailed information of the materials and resources is included in Table 1. All animal experimental procedures were approved by the Institutional Animal Care and Use Committee of the Korea Brain Research Institute (IACUC-2017-0047). All experiments were carried out in accordance with the approved guidelines and regulations.

**Table 1:** Key resource table.

Reagent type or resources	Source or reference	Identifiers	Additional information
**Antibodies**			
Rabbit polyclonal anti-Rab5	Abcam	ab13253	IF 1:200
Mouse monoclonal anti-Rab7	Abcam	ab50533	IF 1:200
Rabbit polyclonal anti-Rab11	Santa Cruz Biotechnology	sc-9020	IF 1:200
Rabbit polyclonal anti-Lamp1	Abcam	ab24170	IF 1:200
Rabbit polyclonal anti-chromograninB	Abcam	ab12242	IF 1:400
Rabbit polyclonal anti-Vamp3	Novus	NB300-510	IB 1:5000 IF 1:200
β-Actin (13E5) rabbit mAb (HRP- conjugated)	Cell Signaling Technology	5125	IB 1:10,000
HRP-conjugated anti-rabbit antibody	Bio-Rad	1706515	IB 1:10,000
Goat anti-mouse IgG (H + L) Alexa Fluor 488	Thermo Fisher Scientific	A11029	IF 1:200
Goat anti-rabbit IgG (H + L) Alexa Fluor 488	Thermo Fisher Scientific	A11034	IF 1:200
Goat anti-rabbit IgG (H + L) Alexa Fluor 568	Thermo Fisher Scientific	A11011	IF 1:200
**Virus strains and DNA**			
pLKO.1-puro eGFP shRNA control target sequence: TACAACAGCCACAACGTCTA	Sigma-Aldrich	SHC005V	
shTrkB #1 (shRNA-pLKO.1-hPGK-puro-CMV-tGFP) target sequence: CATTCCAAGTTTGGCATGAAA	Sigma-Aldrich	SHCLNV-NM_008745	TRCN0000023703
shTrkB #2 (shRNA-pLKO.1-hPGK-puro-CMV-tGFP) target sequence: CCACGGATGTTGCTGACCAAA	Sigma-Aldrich	SHCLNV-NM_008745	TRCN0000023701
pEGFP-hVAMP3	Addgene	42310	Gift from Thierry Galli
pCMV-TeLC-P2A-EYFP	This paper	N/A	
pCAG-EGFP	Addgene	89684	Gift from Wilson Wong
**Chemicals and solutions**			
HEPES	Thermo Fisher Scientific	15630080	
HBSS	Thermo Fisher Scientific	14170112	
Trypsin–EDTA (0.25%), phenol red	Thermo Fisher Scientific	25200056	
B-27 Supplement (50×), serum-free	Thermo Fisher Scientific	17504044	
Penicillin–streptomycin (5000 U/mL)	Thermo Fisher Scientific	15070063	
Neurobasal medium	Thermo Fisher Scientific	21103049	
Polyethylenimine (PEI)	Sigma-Aldrich	P3143	
Fetal bovine serum, ultra-low IgG	Thermo Fisher Scientific	16250-078	
DMEM	HyClone	SH30243.01	
HBEGF	Sigma-Aldrich	E4643	
Lipofectamine 2000	Thermo Fisher Scientific	11668027	
Lipofectamine RNAiMax	Thermo Fisher Scientific	13778100	
TRIzo LS reagent	Thermo Fisher Scientific	10296028	
SuperScript III reverse transcriptase	Thermo Fisher Scientific	18080044	
Human BDNF-biotin	Alomone Labs	B-250-B	
Bovine serum albumin (BSA), biotinylated	Vector Laboratories	B-2007	
Qdo 655 streptavidin conjugate	Thermo Fisher Scientific	Q10121MP	
QSY 21 carboxylic acid, succinimidyl ester	Thermo Fisher Scientific	Q20132	
4% Paraformaldehyde solution (PFA)	Biosesang	PC2031-100-00	
Normal goat serum	Jackson Immunoresearch	005-000-121	
MitoTracker red CMXRos	Thermo Fisher Scientific	M7512	
Mounting medium with DAPI	Vector Laboratories	H-1200-10	
Adenosine 5′-triphosphate magnesium salt (ATP)	Sigma-Aldrich	A9187	
Ionomycin calcium salt	Sigma-Aldrich	I3909	
BAPTA-AM	Sigma-Aldrich	A1076	
**Strains and cell lines**			
Mouse: C57BL/6N	Koatech Co., Korea	N/A	
Cell line: C8-D1A	ATCC	CRL-2541	
**Oligonucleotides**			
TeLC-P2A-EYFP forward: CCCAAGCTTGCCACCATGCCGATCACCATCAACAACT	This paper	N/A	For subcloning
TeLC-P2A-EYFP reverse: CCGCTCGAGTTACTTGTACAGCTCGTCCATG			
siSCR-sense: UAAGGCUAUGAAGAGAUACUU	Ref.^21^	N/A	
siSCR-antisense: AAGUAUCUCUUCAUAGCCUUA			
siVamp3 #1-sense: CCAAGUUGAAGAGAAAGTAUU	TRC Library Database	TRCN0000110516	https://portals.broadinstitute.org/gpp/public
siVamp3 #1-antisense: AAUACUUUCUCUUCAACUUGG			
siVamp3 #2-sense: GUCAAUGUGGAUAAGGUGUUA		TRCN0000110517	
siVamp3 #2-antisense: UAACACCUUAUCCACAUUGAC			
siVamp3 #3-sense: AGGUGCCUCGCAGUUUGAAAC		TRCN0000436473	
siVamp3 #3-antisense: GUUUCAAACUGCGAGGCACCU			
siVamp3 #4-sense: UCAGUGUCCUGGUGAUCAUUG		TRCN0000311406	
siVamp3 #4-antisense: CAAUGAUCACCAGGACACUGA			
TrkB-sense: GCGCTTCAGTGGTTCTACAA	This paper	N/A	For RT-PCR
TrkB-antisense: TTGGGTTTGTCTCGTAGTC	Ref.^22^	N/A	
β-actin-sense: TGTTACCAACTGGGACGACA	Ref.^23^	N/A	
β-actin-antisense: GGGGTGTTGAAGGTCTCAAA			
**Software and algorithms**			
ImageJ (ver. 2.1.0/1.53c)	https://imageJ.nih.gov/ij		
Prism 8.0	GraphPad	N/A	
Others			
100 μm cell strainer	BD Falcon	352360	
Amicon ultra-0.5 centrifugal filter unit	Sigma-Aldrich	UFC510096	
Glass-bottom dish	SPL	101350	

*HRP* horseradish peroxidase, *mAB* monoclonal antibody, *HBSS* Hank’s balanced salt solution, *DMEM* Dulbecco’s modified eagle medium, *HBEGF* heparin binding EGF like growth factor, *DAPI* 4’,6-diamidino-2-phenylindole, *BAPTA* 1,2-bis(*o*-aminophenoxy)ethane-*N,N,N′,N*′-tetraacetic acid, *C8-D1A* mouse astrocyte type 1 clone cell line, *TeLC* tetanus toxin light chain.

### Primary astrocyte culture

We utilized an AWESAM astrocyte culture protocol as reported previously^19^ with minor modifications to acquire cultured astrocytes that had an in vivo-like morphology. Cortical astrocytes were prepared from embryos from wild-type C57BL/6 mice on days E17-18. Cortices were dissected in dissection medium (10 mM HEPES in HBSS) at 4 °C and then incubated in 0.25% trypsin-EDTA in a 37 °C water bath for 20 min with gentle inversion every 5 min. After trypsinization, the tissue was washed in dissection medium at 4 °C five times and then triturated with 1 ml of NB+ medium (2% B-27 supplement, 2 mM GlutaMax, 5000 U/ml penicillin and 5000 µg/ml streptomycin in neurobasal medium). Dissociated cells were filtered through a cell strainer and plated on 0.04% polyethylenimine (PEI)-coated cell culture dishes (4 × 10^6^ cells/60 mm dish) in culture media (10% FBS, 5000 U/ml penicillin and 5000 µg/ml streptomycin in DMEM). Seven days after plating the dissociated cells, the dishes were shaken at 110 rpm for 6 h. The cells were then washed with 1× PBS three times, treated with 0.25% trypsin, and plated on 0.04% PEI-coated glass-bottom dishes (3 × 10^4^ cells/dish) or 18 mm coverslips in a 12-well plate (1 × 10^4^ cells/well) in NB+ medium containing HBEGF (50 µg/ml).

### Transfection of DNA and siRNAs

DNA and siRNA constructs were transfected into cultured astrocytes with Lipofectamine 2000 at 10–11 DIV according to the manufacturer’s protocol. To generate pCMV-TeLC-P2A-EYFP, TeLC-P2A-EYFP fragments were amplified from pAAV-hSyn-FLEX-TeLC-P2A-EYFP-WPRE (Addgene plasmid #135391) with a specific set of primers (Key Resources Table) and then subcloned into a pcDNA3.1 vector by using the HindIII-XhoI site.

To screen *Vamp3* siRNA, C8-D1A (mouse type 1 astrocyte cell line) cells were cultured in DMEM supplemented with 10% FBS at 37 °C under 5% CO_2_. Each siRNA (100 nM) was transfected into C8-D1A cells using RNAi Max according to the manufacturer’s protocol. Two days after transfection, samples were analyzed by western blotting with an anti-Vamp3 primary antibody or β-actin-HRP and HRP-conjugated anti-rabbit secondary antibody. The screening of Vamp3 siRNAs revealed that siVamp3 #1 effectively diminished the level of endogenous Vamp3 (Fig. S2). Therefore, only siVamp3 #1 was employed in the experiments.

*TrkB*-targeting shRNA lentiviral particles were purchased from Sigma (shRNA-pLKO.1-hPGK-puro-CMV-tGFP). The shRNA target sequences are described in the Key Resources Table. To assess the knockdown efficiency of *TrkB* shRNA, cortical neurons from E17-18 C57BL/6 mouse embryos were cultured. Each Lenti-shTrkB particle was transduced into cortical neurons at 5 DIV. Three days after transduction, total RNA was extracted using TRIzol reagent. Each RNA sample (0.3 µg) was reverse transcribed into cDNA by using SuperScript III reverse transcriptase. To determine the reduction in TrkB RNA levels, PCR was performed using TrkB and β-actin primers. Because shTrkB #1 reduced the level of endogenous TrkB more effectively than shTrkB #2 (Fig. S1), only shTrkB #1 was used in the experiments.

### Immunocytochemistry

To determine the localization of QD-BDNF, cultured astrocytes were incubated with 2 nM QD-BDNF for 20 min and then fixed with 4% paraformaldehyde (PFA). For immunostaining, the cells were permeabilized with 0.1% Triton X-100 for 10 min and then blocked with 5% normal goat serum for 1 h at room temperature. After blocking, the cells were incubated with anti-Rab5, anti-Rab7, anti-Rab11, anti-Lamp1, anti-Vamp3, or anti-chromograninB for 1 h and then incubated with an anti-Alexa 488 secondary antibody for 1 h at room temperature.

### QD imaging

For monitoring endocytic BDNF, 50 nM biotinylated mature BDNF (bt-BDNF) or 50 nM biotinylated bovine serum albumin (bt-BSA) was incubated with 50 nM streptavidin-conjugated quantum dot 655 (st-QD655) at 4 °C overnight at a ratio of 2:1. QD-BDNF or QD-BSA was then filtered with a 100 kDa Amicon filter to remove unconjugated mBDNF, BSA, or QDs, and 1% BSA containing PBS was added to the filtrates. Astrocytes were incubated with QD-BDNF or QD-BSA on 12–13 DIV, and the medium was then replaced with an extracellular solution (in mM; 119 NaCl, 2.5 KCl, 20 HEPES, 2 CaCl_2_, 30 glucose, and 2 MgCl_2_, pH 7.4) containing 4 µM QSY21. Time-lapse images were taken by using a confocal laser scanning microscope (TCS SP8, Leica) at a 1 Hz rate using a 63× oil objective. ATP (100 µM) or ionomycin (1 µM)-containing extracellular solution was added to stimulate the astrocytes. QD655 fluorescence was excited with a 561 nm laser and assessed with a HyD (hybrid) detector in the range of 650–695 nm.

### Image and statistical analyses

**Figure 1:**
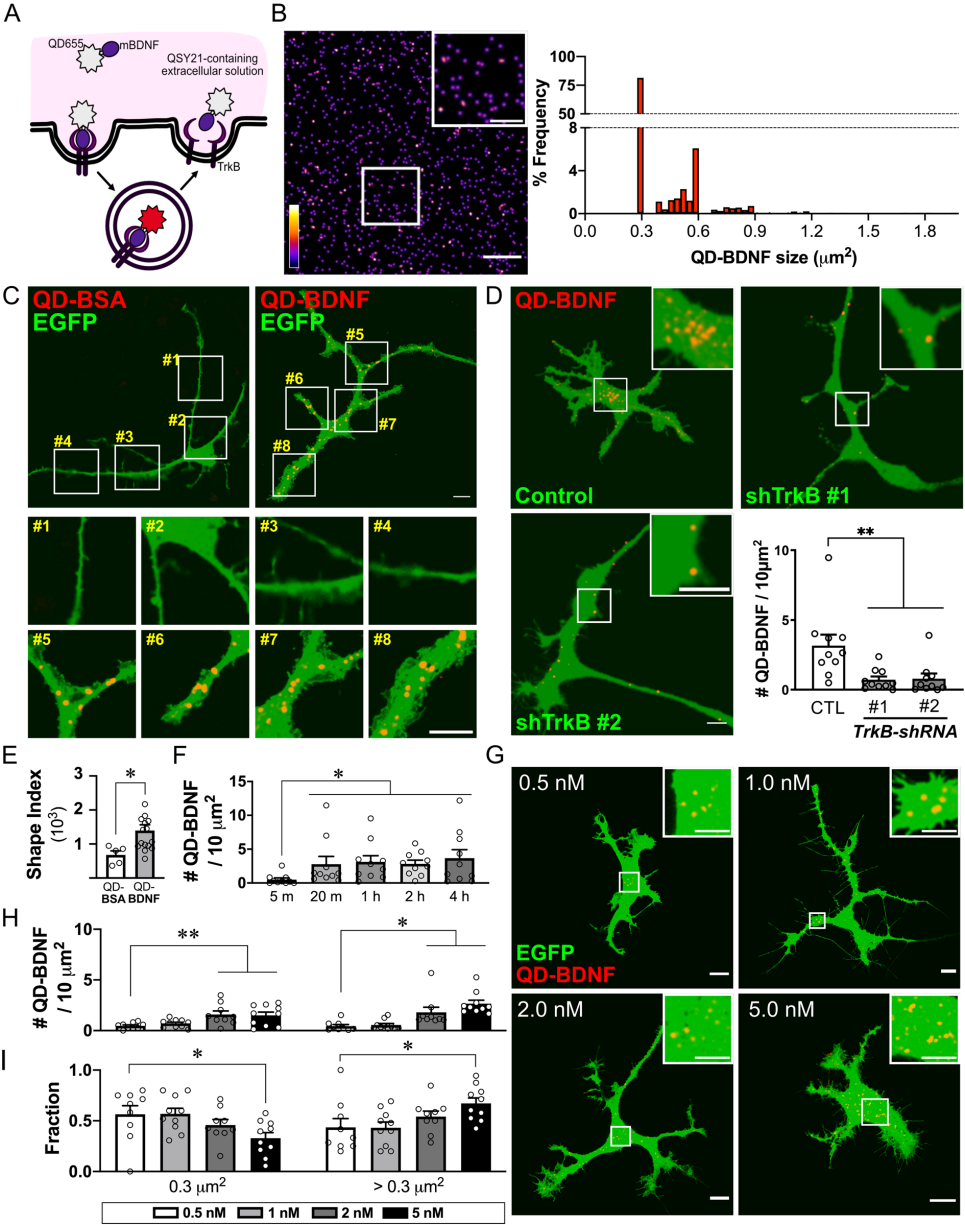
QD-BDNF as a tool for monitoring endocytic BDNF in astrocytes. **(A)** Schematic diagram of biotinylated mBDNF conjugated with streptavidin-QD655 (QD-BDNF). (**B)** Left: Representative fluorescence image of purified QD-BDNFs (2 nM). Scale bar = 10 µm, inset scale bar = 5 µm. Right: distribution of the 2D sizes of QD-BDNF particles (1073 particles from 40 cells). (**C)** Representative images of EGFP-expressing astrocytes treated with QD-BSA or QD-BDNF. Scale bar = 10 µm. Below: magnified views of the indicated locations (numbers). Scale bar = 10 µm. (**D)** Representative images of endocytic QD-BDNFs in astrocytes expressing scrambled shRNA (Control), *TrkB*-shRNA #1 (shTrkB #1), or #2 (shTrkB #2). Scale bar = 10 µm. Inset: magnified view of the indicated location (white box). Scale bar = 5 µm. Bar graphs: average QD-BDNF densities under each condition. **P < 0.01. *N* = 10 cells for each group. **E.** Average shape indices of QD-BSA- and QD-BDNF-treated astrocytes. *P < 0.05. *N* = 5 or 16 cells. (**F)** Average QD-BDNF densities at each incubation time treated with 2 nM of QD-BDNF. *P < 0.05. *N* = 10 cells in each condition. **G.** Representative images of astrocytes treated with 0.5, 1, 2, or 5 nM QD-BDNF. Scale bar = 10 µm, inset scale bar = 5 µm. (**H)** Average QD-BDNF densities with minimum (0.3 µm^2^) or larger sizes (> 0.3 µm^2^). *P < 0.05, **P < 0.01. (**I)** Average fractions of QD-BDNF with minimum or larger sizes among total intracellular QD-BDNF. *P < 0.05. *N* = 9–10 cells in each condition. The quantification of QD-BDNF particle numbers and the shape index of astrocytes were determined by using ImageJ/FIJI software (Ver. 2.1.0/1.53c, NIH).

Image processing and analysis were performed using ImageJ/FIJI software (NIH, USA). To analyze the kinetics or secretion of BDNF particles, regions of interest (ROIs) of astrocytic processes were manually selected and linearized. The linearized time-lapse images were transformed into kymographs using the KymographBuilder plugin in ImageJ/FIJI. After extracting the X and Y coordinate data for each particle from the kymograph, the direction, distance, and velocity were determined. Immobile or QD-BDNF particles in the stationary mode were defined when the particles showed the travel distance less than the diameter of a single QD-BDNF particle (~ 0.6 µm). Trafficking of QD-BDNF particles over 0.6 µm were categorized as the anterograde or retrograde transport, depending on the direction of the particle transports. The complete disappearance of QD-BDNF fluorescence was defined as QD-BDNF exocytosis. The percentage of QD-BDNF secretion was determined by dividing the number of secreted QD-BDNF particles with the total number of QD-BDNF particles on the kymograph (number secreted QD-BDNF particles/all QD-BDNF particles × 100 (%)). To calculate colocalization ratios of QD-BDNF particles, images with QD-BDNF particle were segmented and transformed to the binary images to identify the region of interests (ROIs) of all observed QD-BDNF particles. A total number of QD-BDNF particles was derived from the total number of ROIs in these images. Next, colocalized QD-BDNF particles with vesicle markers were determined when more than 80 % area of the ROI was occupied by the fluorescence signal of vesicle markers. This 80% threshold was based on our confocal imaging conditions as follows: using the oil-immersed 63× lens (numerical aperture (NA) = 1.4) and the 561 nm excitation laser, the approximate lateral resolution of our confocal imaging was about 150 nm (d = 0.37λ/NA, according to the Abbe diffraction limit; λ = 561 nm, NA = 1.4). Because this lateral resolution of our confocal imaging was about 25% of the diameter of single QD particles (the diameter of the single QD particle = ~ 600 nm; Fig. 1), QD-BDNF particles showing 75% or more overlap of their area with fluorescence signals of vesicle markers may be considered as ‘colocalized’ with the tested marker. In Fig. 3B,D, we counted the number of QD-BDNF particles showing 80–100% overlap of their areas with vesicle markers as the number of colocalized QD-BDNF. In Fig. 3D above, colocalized Vamp3-EGFP signals were defined as ones showing 100% overlap of their areas with tested vesicle markers. The colocalization ratio was calculated by the following equation:

$${\text{Colocalization}} \,\, {\text{ratio}}= ({\text{number}} \,\,{\text{of}} \,\, {\text{colocalized}} \,\, {\text{particles}} \,\, {\text{with}} \,\, {\text{vesicular}} \,\, {\text{markers}})/({\text{total}} \,\, {\text{number}} \,\, {\text{ of}} \,\,{\text{particles}}).$$

To analyze the structural complexity of astrocytes induced by BDNF, 2 nM of QD-BDNF was treated for 20 min. The morphological complexity of astrocytes was defined by the shape index (SI; cell perimeter^2^/area – 4π). It is known that greater SI values well correspond to increased complexity of cell morphology, but perfect circles show SI = 0^12,20^.

Statistical analyses were performed using Prism 8.0 software (GraphPad). Statistically significant differences between two groups were determined using Student’s unpaired t-test, and three or more groups were compared using one-way ANOVA with Dunnett’s multiple comparisons test. The Kolmogorov-Smirnov test was used to examine the statistical significance of the percentages of cumulative distribution between the two groups. All data were from three independent batches of cultured astrocytes and are indicated as the mean ± standard error of the mean (SEM).

## Results

### Monitoring endocytic BDNF in cultured astrocytes using QD-BDNF

**Figure 2:**
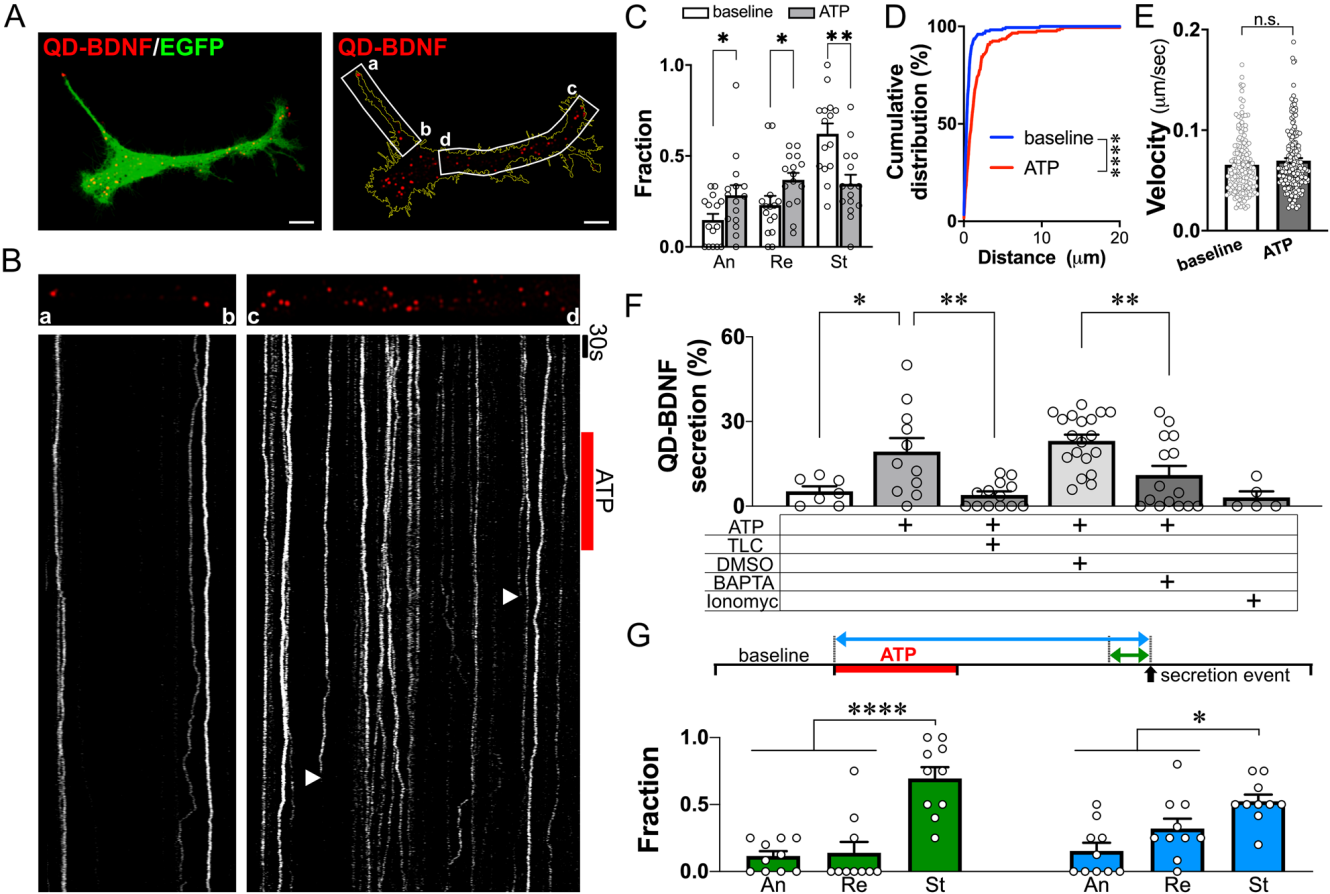
ATP stimulation results in the Ca^2+^-dependent exocytosis of endocytic BDNF. **(A)** Left: Representative fluorescence image of EGFP-expressing astrocytes containing QD-BDNF. Right: QD fluorescence image of the cell in (**A)**. Yellow line: cell boundary determined by EGFP signals. White boxes: linearized segments used to generate the kymographs in (**B**) Scale bar = 10 µm. (**B)** Representative kymographs indicated in (**A)** generated by using ImageJ/FIJI software (Ver. 2.1.0/1.53c, NIH). Red bar: ATP (100 µM) treatment. Arrow heads: disappearances of QD-BDNF fluorescence. (**C)** Average QD-BDNF fractions showing immobility (St) or anterograde (An)/retrograde (Re) transport. *P < 0.05, **P < 0.01. (**D)** Cumulative distributions of the QD-BDNF transport distances at baseline and after ATP stimulation (ATP). ****P < 0.0001. (**E)** Average velocities of mobile QD-BDNF particles. n.s., not significantly different. *N* = 173 particles from 14 cells for each group. (**F)** Average percentages of secreted QD-BDNF. TLC: tetanus toxin light chain. Ionomyc: ionomycin (1 µM). *P < 0.05, ** P < 0.01. *N* = 5–11 cells for each group. (**G)** Average secreted QD-BDNF fractions showing immobility or An/Re transport before exocytosis. Green: 60 s before exocytosis. Blue: immediately prior to ATP treatment. *P < 0.05, ****P < 0.0001.

To directly monitor endocytic BDNF in astrocytes, we utilized biotinylated recombinant mature BDNF directly associated with streptavidin-QDs as described previously (ref.^7^; see Methods for detailed information). With this method, the fluorescence of the extracellular QD-conjugated mature BDNF complex (QD-BDNF; Fig. 1A) could be cancelled by a hydrophilic fluorescence quencher, QSY21 (4 µM), in the extracellular media, but QD-BDNF fluorescence was recovered after endocytosis (Fig. 1A,C). Under our imaging conditions, the smallest and most observable two-dimensional size of purified QD-BDNF was approximately 0.3 µm^2^, indicating a single QD-BDNF particle (Fig. 1B). The intracellular uptake of QD-BDNF particles into astrocytes was mediated by receptor-mediated endocytosis, as (1) QD-BSA treatment resulted in no intracellular QD particles (Fig. 1C), and (2) the number of intracellular QD-BDNF particles (Fig. 1D) from astrocytes was significantly reduced by shRNA-mediated genetic knockdown (KD) of *TrkB* expression (Fig. S1). Moreover, our QD-BDNF particles were bioactive, because cultured astrocytes showed more complex morphology after QD-BDNF treatment (Fig. 1E), consistent with a previous report^12^. Since astrocytic TrkB.T1-dependent structural complexity is important for the structural and functional maturation of astrocytes^12^, QD-BDNF uptake under our conditions appeared to be mediated by TrkB.T1.

We next explored the ideal concentration and incubation time for the QD-BDNF treatment of cultured astrocytes to track single QD particles. QD-BDNF (0.5–5 nM) was applied to cultured astrocytes for 5 minutes (min) up to 4 h. Treatment with 2 nM QD-BDNF for 20 min resulted in most density and fraction of intracellular single QD-BDNF particles (Fig. 1F–I), and all QD-BDNF tracking and secretion experiments were therefore carried out under this condition.

### ATP triggers the transport and secretion of endocytic BDNF in astrocytes

We next monitored intracellular QD-BDNF particles in astrocytes to investigate the transport and secretion of endocytic mBDNF. Since astrocytes can be stimulated by extracellular ATP due to the expression of diverse P2 receptors^24^, 100 µM ATP was added to QD-BDNF-containing astrocytes expressing EGFP (Fig. 2A,B) to induce the transport and secretion of QD-BDNF. Most QD-BDNF particles remained immobile (stationary mode) before ATP treatment (Fig. 2C). However, ATP stimulation triggered either the anterograde or retrograde transport of QD-BDNF (Fig. 2C), leading to an increase in the distance of QD-BDNF trafficking (Fig. 2D). No ATP-induced changes in speeds of QD-BDNF transport were detected (Fig. 2E). These results suggest that the transport of endocytic BDNF is dependent on ATP-induced intracellular signaling.

We next assessed whether ATP stimulation evokes endocytic BDNF release in astrocytes. The exocytosis of endocytic QD-BDNF could be detected by the disappearance of QD-BDNF fluorescence due to the exposure of QD-BDNF to the QSY21 quencher via opened vesicle pores^7^. Despite a few spontaneous QD-BDNF exocytosis events (5.28 ± 1.76%), QD-BDNF exocytosis was significantly increased (19.37 ± 4.75%; Fig. 2F) after the ATP treatment, consistent with another study^25^. This ATP-induced QD-BDNF secretion was abolished by the expression of the tetanus toxin light chain (TLC) in astrocytes (Fig. 2F), supporting the idea that endocytic BDNF release is SNARE-dependent. Ca^2+ ^signaling seems to play a limited role in endocytic BDNF secretion, because ATP-induced QD-BDNF secretion was partially reduced by the chelation of intracellular Ca^2+^, but a direct Ca^2+^ elevation by the ionomycin treatment failed to trigger QD-BDNF secretion (Fig. 2F). These results suggest that cooperative actions of other mechanisms with Ca^2+^ signaling are required for the full exocytosis of endocytic BDNF-containing vesicles. Finally, as reported in neurons^7^, BDNF secretion events were frequently observed in immobile vesicles before ATP treatment (Fig. 2G), suggesting that the arrival of endocytic BDNF vesicles at secretion sites is a prerequisite for exocytosis events.

### Subcellular localization of endocytic BDNF in astrocytes

**Figure 3:**
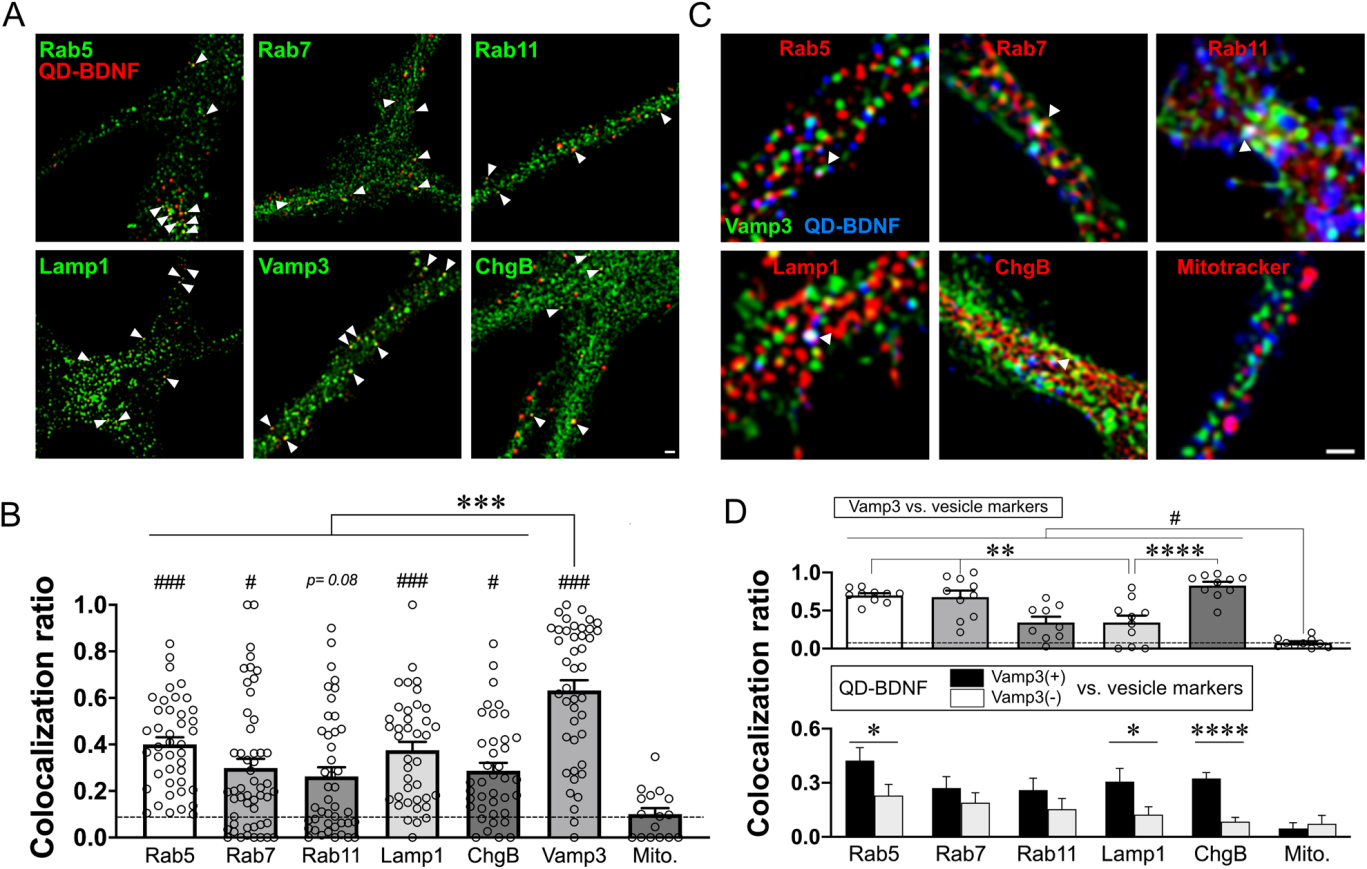
Subcellular localization of endocytic BDNF in cultured astrocytes. **(A)** Representative fluorescence images of the colocalization of QD-BDNF with endogenous vesicular markers. ChgB: chromograninB. Scale bar = 2 µm. White arrowheads: representative colocalization of QD-BDNF with the corresponding markers. (**B)** Average colocalization ratios (# colocalized QD-BDNF/# total QD-BDNF). Dotted line: average colocalization ratio between QD-BDNF and MitoTracker (Mito.; negative control). **P < 0.01 (Vamp3 vs. others), ^##^*P* < 0.01 (Mito. vs. others). *N* = 39–45 cells (1,834–10,531 QD particles) for vesicular markers. (**C)** Representative fluorescence images of the colocalization of QD-BDNF, Vamp3-EGFP, and other vesicular markers. Scale bar = 2 µm. White arrowheads: representative triple colocalization among QD-BDNF, Vamp3-EGFP, and the corresponding vesicular markers. (**D)** Above: average colocalization ratio between Vamp3-EGFP and each vesicular marker. *P < 0.05, **P < 0.01. Below: average colocalization ratio of each vesicular marker with QD-BDNF with Vamp3-EGFP (Vamp3( +)) or without Vamp3-EGFP (Vamp3(−)). **P < 0.01. *N* = 9–10 cells (QD-BDNF particles: 124–481; Vamp3-EGFP puncta: 449–626). Colocalization ratios were determined by using ImageJ/FIJI software (Ver. 2.1.0/1.53c, NIH).

Because endocytosed QD-BDNF showed ATP-induced transport and secretion, we next sought to determine the localization of QD-BDNF after endocytosis. To examine vesicular fractions containing QD-BDNF, immunocytochemistry was performed using antibodies labeling selective vesicular fractions such as Rab5 (early endosomes), Rab7 (late endosomes), Rab11 (recycling endosomes), Lamp1 (lysosomes), and chromograninB (ChgB; secretory granules) (Fig. 3A). Vamp3 was also assessed due to its high expression in astrocytes^15,16^.

QD-BDNF particles were widely detected in all the tested vesicular fractions (Fig. 3A,B). Of note, the colocalization ratio of QD-BDNF with Vamp3 was highest among that with other vesicular markers (Fig. 3B), suggesting that a large portion of internalized BDNF molecules was sorted into Vamp3-positive vesicles. To further characterize the Vamp3-positive QD-BDNF-containing vesicles, additional immunocytochemistry analyses of astrocytes with both QD-BDNF particles and Vamp3-EGFP were performed with vesicular marker antibodies (Fig. 3C). Regardless of whether QD-BDNF particles were detected, Vamp3-positive vesicles were enriched in vesicles containing Rab5, Rab7, or ChgB (Fig. 3C,D). However, Vamp3-positive vesicles with QD-BDNF were more colocalized with Rab5-, Lamp1-, or ChgB-positive vesicles than Vamp3-negative ones (Fig. 3C,D). Given that astrocytic Vamp3-containing vesicles are implicated in the exo- and endocytotic cycling of endosomes^16^, our results suggest that Vamp3 participates in endocytic BDNF recycling in astrocytes.

### Vamp3 is required for ATP-induced endocytic BDNF secretion from astrocytes

**Figure 4:**
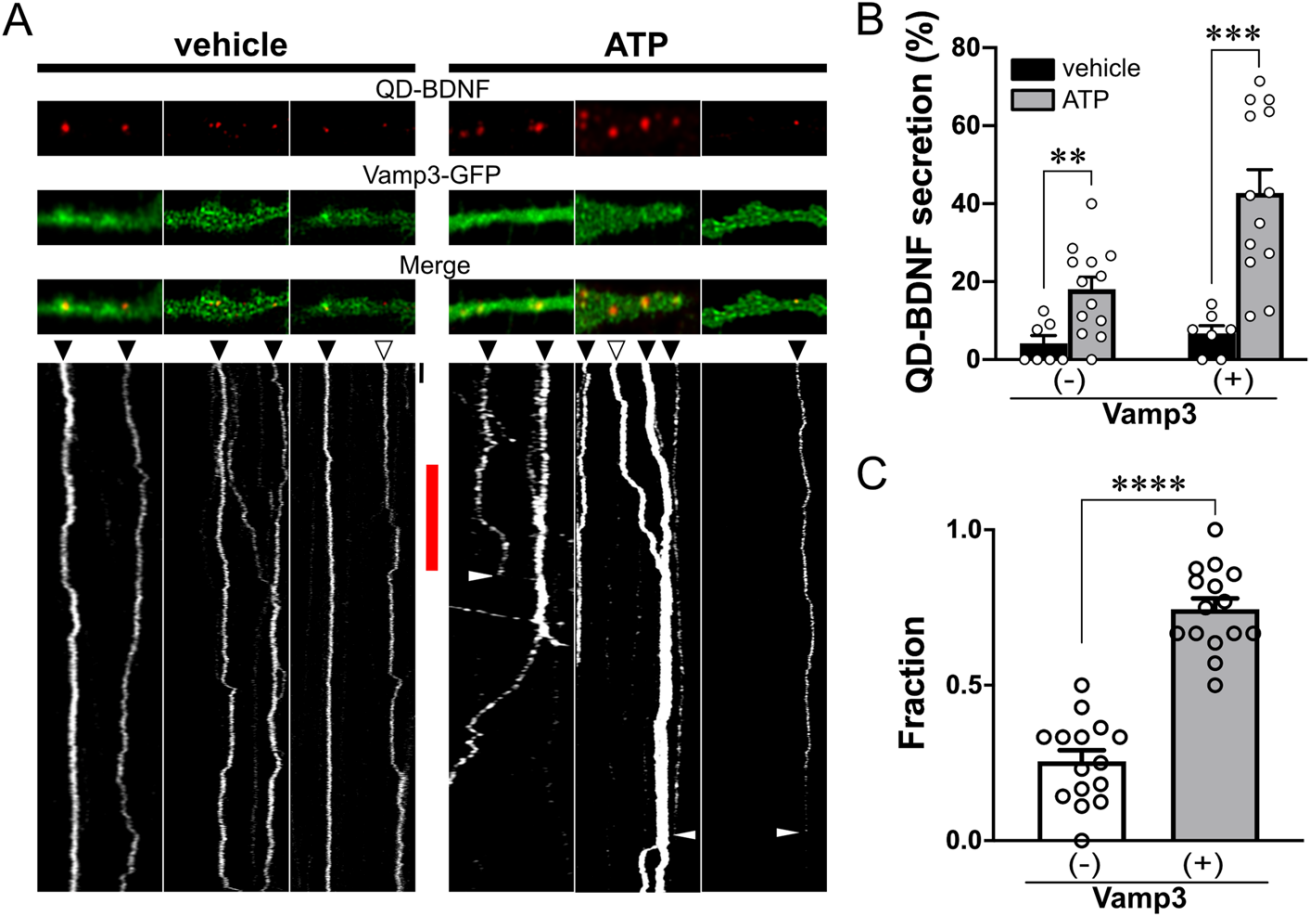
ATP-induced secretion of endocytic BDNF from Vamp3-containing vesicles. **(A)** Representative fluorescence images of astrocytic processes containing QD-BDNF and Vamp3-EGFP. The kymograph was generated by using ImageJ/FIJI software (Ver. 2.1.0/1.53c, NIH). Black arrow heads: Vamp3-positive QD-BDNF particles. Empty arrowheads: Vamp3-negative QD-BDNF particles. Red bar: ATP treatment. White sharp arrowheads: disappearance of QD-BDNF particles. Black bar = 30 s. (**B)** Average percentages of ATP-induced QD-BDNF secretion events from vesicles with ( +) or without Vamp3 (−). **P < 0.01, ***P < 0.001. N = 15 cells (vehicle = 162; ATP = 333 QD particles). (**C)** Average fractions of secreted QD-BDNF particles with ( +) or without Vamp3 (−) among total secreted QD-BDNFs. ****P < 0.0001. *N* = 15 cells (107 secreted QD particles).

Since our results showed that endocytic BDNFs were enriched in Vamp3-containing astrocytic vesicles (Fig. 3), ATP-induced BDNF secretion may frequently occur at Vamp3-positive vesicles. We thus compared the fraction of QD-BDNF particles displaying the exocytosis event from Vamp3 ( +) vesicles to that from Vamp3-negative (−) vesicles (Fig. 4A). Few very spontaneous QD-BDNF secretion events were observed regardless of the presence of Vamp3 in QD-BDNF-containing vesicles (Fig. 4B), indicating that spontaneous endocytic BDNF release does not involve Vamp3. ATP-induced QD-BDNF secretion events was also observed from both Vamp3-positive and Vamp3-negative vesicles (Fig. 4B), but QD-BDNFs in Vamp3-positive vesicles were secreted more frequently than those in Vamp3-negative vesicles (Fig. 4C). Despite the possible effect of Vamp3-EGFP overexpression on distribution of endocytic QD-BDNF, these results propose the involvement of Vamp3-positive vesicles in ATP-induced endocytic BDNF secretion.

**Figure 5:**
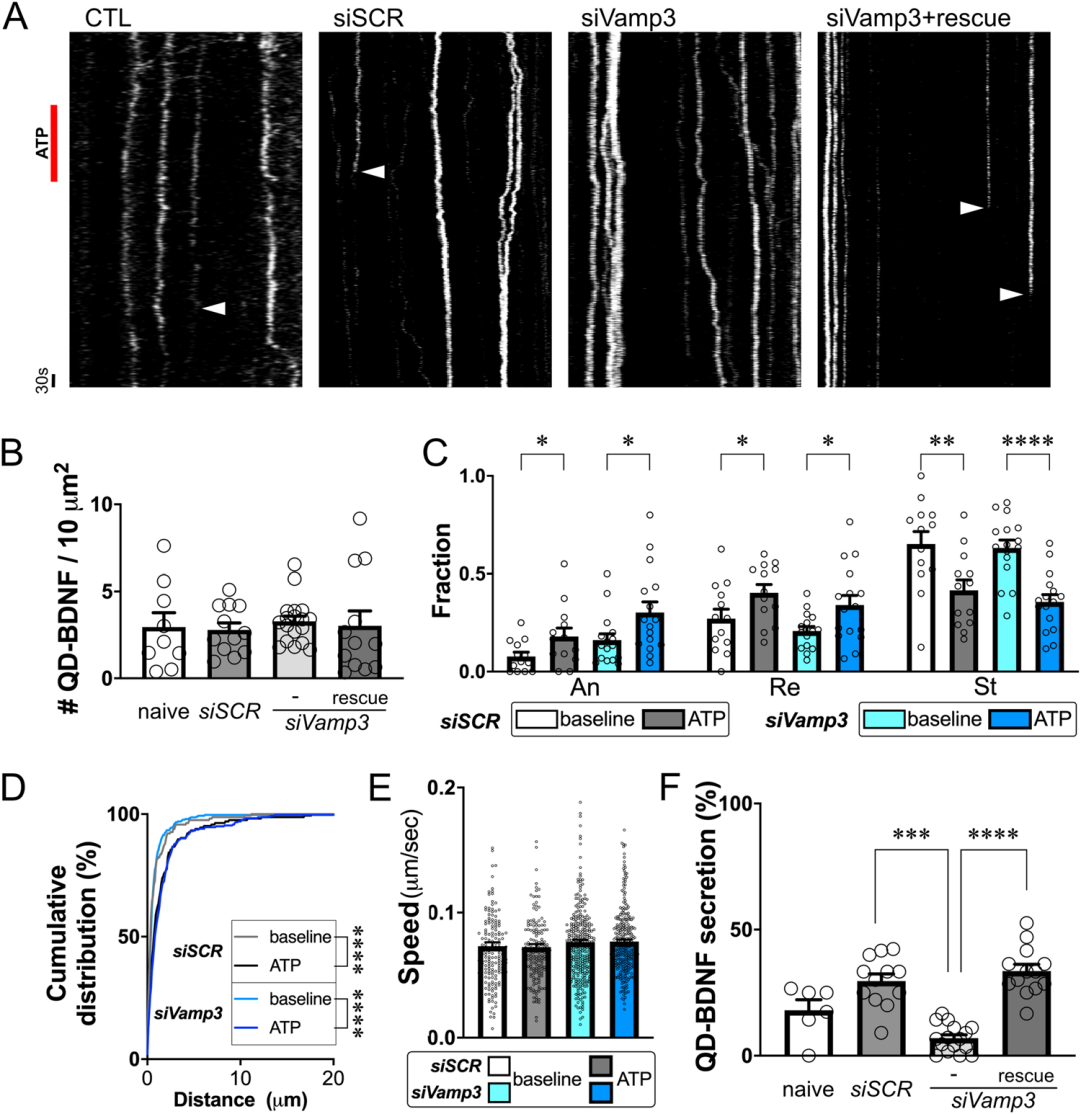
Vamp3 is necessary for ATP-induced endocytic BDNF secretion. **(A)** Representative QD-BDNF kymographs generated by using ImageJ/FIJI software (Ver. 2.1.0/1.53c, NIH), from astrocytes with GFP- (CTL), *siSCR*-, *siVamp3*-, or *siVamp3* + human *Vamp3* (*siVamp3* + rescue). White arrowheads: disappearance of QD-BDNF particles. (**B)** Average intracellular QD-BDNF densities under each condition. *N* = 9–18 cells. The quantification of QD-BDNF particle numbers was determined by using ImageJ/FIJI software (Ver. 2.1.0/1.53c, NIH). (**C)** Average QD-BDNF fractions showing immobility (St) or anterograde (An)/retrograde (Re) transport. *P < 0.05, **P < 0.01, ****P < 0.0001. *N* = 10–16 cells. (**D)** Cumulative distributions of the QD-BDNF transport distances at baseline and after ATP stimulation (ATP) in each group. ****P < 0.0001. (**E)** Average velocities of QD-BDNF transport. *N* = 165 and 302 particles for the *siSCR* and *siVamp3* groups, respectively. **F.** Average percentages of QD-BDNF secretion events after the indicated treatments. **P < 0.01, ***P < 0.001, ****P < 0.0001. N of tested cells (with QD particle number): naïve = 6 (102), *siSCR* = 12 (197), *siVamp3* = 16 (304), *siVamp3* + rescue = 13 (187).

Next, we tested whether Vamp3 directly participates in endocytic BDNF exocytosis by using the siRNA mediated KD method (Fig. S2C). We first assessed whether the endocytosis or transport of QD-BDNF was affected by *Vamp3* KD (Fig. 5A–E). *Vamp3* KD failed to alter the endocytosis (Fig. 5B) or ATP-induced antero- or retrograde transport of QD-BDNF (Fig. 5C–E). By contrast, astrocytes with *Vamp3* KD showed significantly reduced ATP-triggered QD-BDNF secretion to ~ 76% (% QD-BDNF secretion: siSCR = 29.61 ± 2.84 vs. siVamp3 = 6.92 ± 1.36; Fig. 5F). This reduced QD-BDNF exocytosis was successfully restored by the delivery of the siRNA-insensitive *Vamp3* construct together with *Vamp3* siRNAs (Fig. 5F). Together, these results indicate that Vamp3 selectively controls endocytic BDNF exocytosis in astrocytes.

## Discussion

In this work, we showed the direct uptake and recycling of mBDNF in astrocytes by utilizing QD-BDNF as a proxy for the extracellular BDNF protein. After secreted from source cells, neurotrophin proteins seem to be internalized by binding to corresponding Trk receptors on nearby target cells, but direct monitoring of endogenous neurotrophin has been hampered due to their relatively low concentration in live cells. Because QD is a fluorescent nanoparticle with an excellent photostability and could stably tracked in live cells with a high signal-to-noise ratio, the QD-linked neurotrophin sensor has been widely used to examine the transport and activity-dependent secretion of neurotrophin-containing endosomes in live cells^7,17,18^. Using QD-linked mBDNF, a previous study founds TrkB-dependent mBDNF internalization, as well as complexin 1/2 (Cpx1/2)/synaptotagmin 6 (Syt6)-dependent re-secretion of endocytic mBDNF^7^. However, it has not examined whether mBDNF is directly internalized and recycled in astrocytes and what molecular mechanisms handle endocytic mBDNF secretion from astrocytes, although astrocytic p75NTR-dependent endocytosis of neuronal proBDNF and its re-secretion were reported^10^.

When treated with purified QD-BDNF particles, there was an increase in the complexity of astrocytic morphology (Fig. 1E), as found from other studies showing TrkB.T1-dependent structural complexity and maturation of astrocytes^11,12^. Given that TrkB-shRNA expression diminished QD-BDNF internalization (Fig. 1D), QD-linked mBDNF endocytosis and morphological changes seem to be mediated by TrkB.T1. Because ATP stimulation of astrocytes was sufficient for triggering SNARE-dependent release of endocytic QD-BDNF (Fig. 2F), our study proposes that neuronal mBDNF directly takes part in the process of astrocytic modulation of extracellular BDNF concentration, in addition to TrkB.T1-dependent regulation of astrocyte functions.

We revealed that Vamp3 is one of important regulators of ATP-triggered endocytic BDNF secretion. Among all tested vesicular pools, Vamp3-positive vesicles in the fraction of early endosomes, lysosome, or secretory granule contained most endocytic QD-BDNFs (Fig. 3B). However, other vesicular fractions such as Rab7 or Rab11-positive endosomes or other SNARE-containing vesicles may contain a portion of endocytic BDNF, because we found significant colocalization of QD-BDNF in both Vamp3-positive and -negative vesicles with corresponding vesicular markers but no significant colocalization with MitoTrackers (Fig. 3D). Because Vamp3 is an enriched vSNARE in astrocytes^29^ and involved in endosome recycling^16^, it is possible that recycling of endocytic BDNF-containing vesicles in astrocytes requires the role of Vamp3. Indeed, our findings support this notion; we observed the secretion of QD-BDNF by ATP stimulation frequently from Vamp3-EGFP-containing vesicles (Fig. 4). *Vamp3* KD was successful in diminishing ~ 76% of ATP-induced QD-BDNF exocytosis (Fig. 5), supporting the idea that Vamp3 is one of the main regulators for endocytic BDNF secretion. However, neither endocytosis nor transports of QD-BDNF requires Vamp3, as shown by no changes in QD-BDNF uptake and transports by astrocytic *Vamp3* KD (Fig. 5). These results indicate a selective role of Vamp3 in endocytic BDNF release. It is unclear how ATP stimulation of astrocytes caused increased the antero- or retrograde transport of endocytic BDNF-containing vesicles, but modification of vesicle trafficking or sorting by P2 receptor-mediated Ca^2+^ or lipid signaling^26–28^ may be implicated.

Our work also uncovered the complex molecular nature underlying endocytic BDNF secretion from astrocytes. We discovered that chelation of ATP-induced Ca^2+^ elevation partially reduces QD-BDNF exocytosis, whereas a direct increase in intracellular Ca^2+^ concentration cannot evoke QD-BDNF exocytosis (Fig. 2F). These findings imply the requirement of additional signaling pathway for full exocytosis of endocytic BDNF. For example, modification of cAMP concentration through P2 receptor activation^30,31^ or A2 receptors^32^, may influence endocytic BDNF release by activating cAMP-dependent signaling pathways important for vesicle docking or exocytosis^29,33^. Moreover, Vamp3-independent mechanisms may also be implicated in regulating endocytic BDNF release, because we observed a significant number of QD-BDNFs localized in the Vamp3 negative vesicles (Fig. 3) and ATP-triggered QD-BDNF release events from Vamp3 (-) vesicles (Fig. 4B). These findings support the notion that astrocytic mBDNF recycling involves multiple but differential signaling pathways. Additional studies will further explore the other aspects of molecular events regulating BDNF recycling in astrocytes and their physiological functions in synaptic plasticity and cognitive functions.

## Supplementary Information

Supplementary Figures.

## Data Availability

All data generated or analyzed during this study are included in this published article (and its supplementary information files).
