# Supplementary material for: Endocytic BDNF secretion regulated by Vamp3 in astrocytes
Source: Sci Rep. 2021 Oct 27;11:21203. doi: 10.1038/s41598-021-00693-w (PMC8551197; doi:10.1038/s41598-021-00693-w)
Supplement: Supplementary file 1 — Supplementary Figures. [file 41598_2021_693_MOESM1_ESM.docx]

**Endocytic BDNF secretion regulated by Vamp3 in astrocytes**

Jeongho Han^1^, Sungryeong Yoon^2^, and Hyungju Park^1,2*^

^1^Research Group of Neurovascular Unit, Korea Brain Research Institute (KBRI), Daegu, 41062, South Korea

^2^Department of Brain and Cognitive Sciences, DGIST, Daegu, 42988, South Korea

^*^Corresponding author:

Hyungju Park, Ph.D. (phj2@kbri.re.kr)

**Supplementary Figures** (Fig. S1~S2)

S
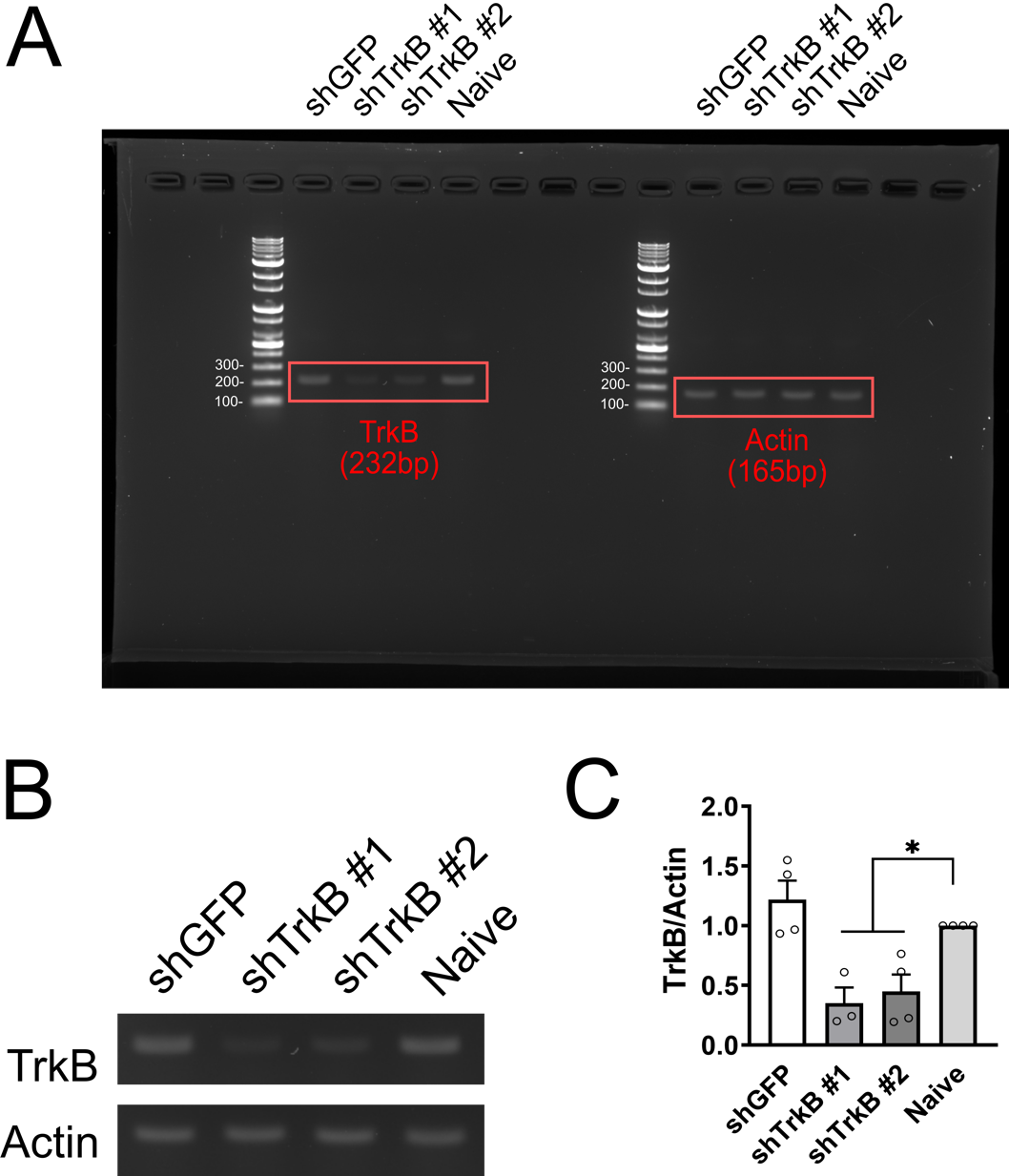


**Figure S1. Genetic KD of TrkB by shRNA delivery**

**A.** Representative full-length RT-PCR results showing the TrkB mRNA (left) and β-actin (actin) mRNA level (right) in shRNA-treated cultured cortical neurons. shGFP: shRNA targeting the EGFP coding sequence. **B.** Cropped RT-PCR results from **A** (red boxes) showing TrkB and actin mRNA levels in each condition. **C.** Normalized TrkB mRNA level in shRNA-treated cultured cortical neurons. **P* < 0.05. *N*= 3 culture batches. The densitometry quantification of the RT-PCR results was determined by using ImageJ/FIJI software (Ver. 2.1.0/1.53c, NIH).


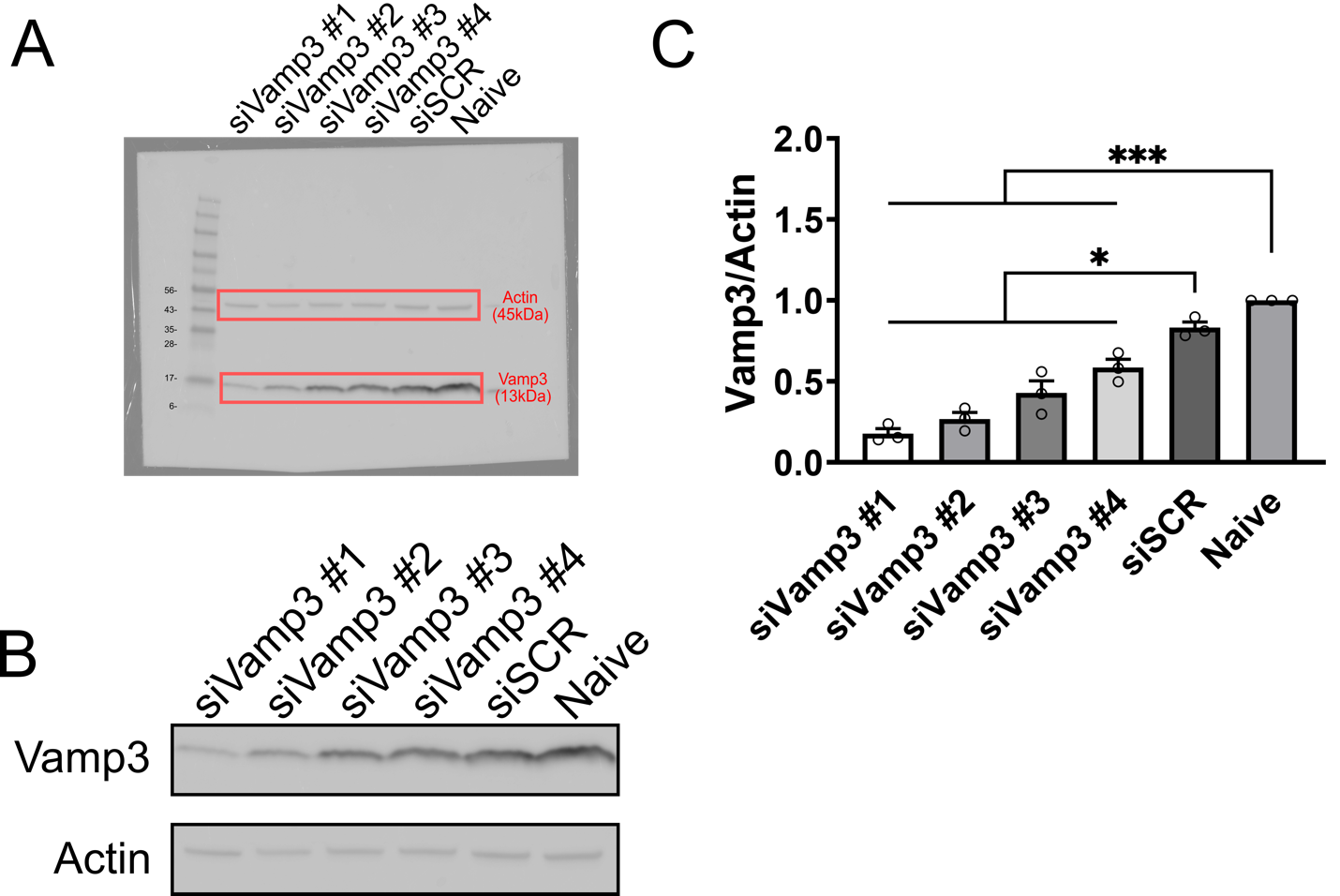


**Figure S2. Genetic KD of Vamp3 by siRNA delivery**

**A.** Representative full-length western blot results showing the β-actin or Vamp3 protein level in siRNA-treated C8-D1A cells. siSCR: scrambled siRNA. **B.** Cropped western blot results from A (red boxes) showing Vamp3 and actin protein levels in each condition. **C.** Normalized Vamp3 protein levels in siRNA-treated C8-D1A cells. **P* < 0.05, ****P* < 0.001. *N*= 3 culture batches. The densitometry quantification of the western blot results was determined by using ImageJ/FIJI software (Ver. 2.1.0/1.53c, NIH).
